# Switching off automatic pilot to promote wellbeing and performance in the workplace: the role of mindfulness and basic psychological needs satisfaction

**DOI:** 10.3389/fpsyg.2023.1277416

**Published:** 2023-12-06

**Authors:** Rachel Guertin, Marie Malo, Marie-Hélène Gilbert

**Affiliations:** ^1^Department of Psychology, Université de Sherbrooke, Sherbrooke, QC, Canada; ^2^Department of Management, Université Laval, Québec, QC, Canada

**Keywords:** psychological wellbeing at work, task performance, trait mindfulness, basic psychological need satisfaction, happy-productive worker thesis, self-determination

## Abstract

**Introduction and method:**

Building on self-determination theory, this study aims to advance the happy-productive worker thesis by examining a sequential mediation linking trait mindfulness to task performance through basic psychological need satisfaction and psychological wellbeing at work. Whereas most of the papers published on the topic stem from USA and Europe, we tested our model in a Canadian sample of 283 French-speaking workers.

**Results:**

Based on structural equation modeling, results show that the three need satisfactions at work mediate the relationship between trait mindfulness and psychological wellbeing at work. Rather than observing a sequential mediation, we find an indirect effect of trait mindfulness on task performance through the satisfaction for one of the basic psychological need (i.e., competence).

**Discussion:**

The present research goes beyond previous studies by exploring a new pair of happy construct-productive criteria alongside an emergent intrapersonal factor contributing to this relationship.

## 1 Introduction

Still portrayed as the Holy Grail of industrial and organizational psychology (Gutiérrez et al., 2020; Arshad et al., 2023; Ghasemy et al., 2023), the happy-productive worker thesis states that the happier people are at work, the better they will perform (Cropanzano and Wright, 1999; Wright and Cropanzano, 2007). Over the past few years, two literature reviews provided a comprehensive overview of the topic, which included over 40 studies, 45 independent samples, and 34,000 participants (Gutiérrez et al., 2020; Sender et al., 2021). The authors’ reviews acknowledge that results remain inconclusive regarding the relationship between happiness and performance. They point out that some pairs of happy construct-productive criterion can either support or reject the much-cited thesis. A possible explanation behind such mixed evidence lies in how happiness and productivity are operationalized. Both reviews emphasize that happiness is defined and assessed through various positive subjective experiences. Positive affect (Gutiérrez et al., 2020; Sender et al., 2021) and job satisfaction (Sender et al., 2021) are the main indicators drawn upon in operationalizing the construct. While positive affect and job satisfaction refer to how people perceive and relate to their work environment, Sender et al. (2021) highlight the multitude of conceptualizations and measures, making a common definition challenging. There is analogous multiplicity in productivity operationalization. Nonetheless, papers analyzed in the studies by Gutiérrez et al. (2020) and Sender et al. (2021) indicate that this concept is largely based on job performance operationalized by behaviors contributing to the organization’s effectiveness (Gutiérrez et al., 2020; Sender et al., 2021).

Among additional key findings drawn from these literature reviews, job satisfaction is positively related to performance in only a third of the articles analyzed (Sender et al., 2021). Conversely, wellbeing has the highest confirmation rate at 82% for the happy-productive worker thesis (Sender et al., 2021), despite being a less-studied indicator of happiness (Gutiérrez et al., 2020; Sender et al., 2021). Taken together, these inconsistent results underline the need for further exploration into pairs of happy construct-productive criterion in pursuing the quest for the Holy Grail. In a similar line, Peiró et al. (2019) called for more attention to be paid to under-explored operationalizations of happiness.

Whereas past studies focused on context-free operationalizations of wellbeing, examining a work-specific indicator would be another step toward a richer understanding of the above relationship. This proposition aligns with Johns (2006, 2017) who argues that studying a construct broadly versus contextualized explains workplace behaviors differently. Accordingly, he recommends considering context to understand better complex organizational phenomena (Johns, 2006, 2017). Additionally, from an empirical perspective, Judge et al. (2017) report that contextualized constructs have better predictive value for specific work behaviors than general variables. These insights underline the need to contextualize wellbeing in terms of workers’ realities rather than using the general conception of this construct, especially knowing that the happy-productive worker thesis is embedded in the organizational environment.

Beyond the search for new pairs of happy construct-productive criterion, researchers and practitioners are also questioning the antecedents of this relationship. Since most studies provide evidence for organizational variables as happiness predictors (e.g., psychological contract, perceived organizational support; Ayala et al., 2017; Joo and Lee, 2017; Fogaça et al., 2021), exploring individual factors offers another way forward. While traits and personal dispositions gained scholars’ attention (e.g., Gutiérrez et al., 2020; Lindberg et al., 2021), researchers primarily focused on the dark side of personality (e.g., anti-social disposition, neuroticism, anger; Gutiérrez et al., 2020). This evidence reflects an unbalanced portrait of antecedents, neglecting individuals’ characteristics that are more under their control and can constructively help them. By the same token, Peiró et al. (2019) and Sender et al. (2021) suggest further investigation into factors contributing to happiness in organizational settings.

In light of these observations, the current research goes beyond previous studies in two ways. First, we advance the scientific understanding of the happiness-performance link by testing an emergent operationalization of wellbeing that accounts for the work context. Specifically, we examine whether psychological wellbeing at work, as an under-explored operationalization of happiness, is related to task performance, one of the most studied criterion variables (Sender et al., 2021). Task performance refers to behaviors recognized by the organization’s formal reward systems and outlined in the job description requirements (Williams and Anderson, 1991). Second, the present work proposes and tests an enriched model building upon a seminal framework shaping the motivational antecedents of wellbeing and performance outcomes. Based on self-determination theory (Deci and Ryan, 1985; Ryan and Deci, 2017), we incorporate an under-examined intrapersonal factor within the happy-productive worker thesis, namely mindfulness – defined as open and receptive awareness of present experiences (Brown and Ryan, 2003). According to one of the theory’s formal propositions (Ryan and Deci, 2017), mindfulness could heighten the potential for satisfying psychological needs, as it may enhance one’s capacity for volitional self-direction in alignment with needs and sources of motivation. Ryan and Deci (2017) state that the fulfillment of basic psychological needs provides the essential conditions for psychological wellbeing and the production of behaviors. Through empirical testing of this expanded model, the current responds to the invitation of Peiró et al. (2019), Gutiérrez et al. (2020), and Sender et al. (2021) by testing a new pair of happy construct-productive criteria and by considering a positive dispositional tendency that places human beings at the center of their experience.

## 2 Theoretical background

### 2.1 Operationalization of wellbeing

Some authors consider wellbeing as the absence of negative symptoms – such as anxiety (e.g., Gu et al., 2023) defined as a natural bodily warning system that is reflected in the feeling of worry and unease over an uncertain outcome (Barnett et al., 2009), or burnout (see Niinihuhta and Häggman-Laitila, 2022 for a review), a prolonged response to chronic emotional and interpersonal stressors on the job (Maslach and Leiter, 2016). Meanwhile, researchers increasingly concur on defining wellbeing by the presence of positive indicators – including positive emotions (e.g., Seligman, 2011) and sense of purpose (e.g., the extent to which individuals felt their lives had meaning, purpose and direction, Ryff, 2013). Several scholars argue that the absence of negative symptoms does not necessarily indicate the presence of wellbeing (e.g., Seligman, 2019; Park et al., 2023; Zhao and Tay, 2023). Back in 2002, Keyes and Lopez shed light on the possibility of concurrently observing negative symptoms and positive indicators of wellbeing among individuals. Nearly 20 years on, a scoping review (Iasiello and van Agteren, 2020) based on 83 peer-reviewed empirical articles emphasizes the distinction between negative and positive subjective experiences. Accordingly, we rely on this perspective to operationalize wellbeing as an indicator of happiness.

When conceived as a positive construct, wellbeing is conceptualized from two main approaches. On one side is the hedonic approach that describes wellbeing in terms of pleasure-seeking, positive emotions, and favorable judgment of satisfaction (Diener, 2000; Cropanzano and Wright, 2001; Fisher, 2010). On the other side is the eudemonic approach that conceptualizes wellbeing as realizing full potential and self-determination, a sense of meaning, and being oneself completely (Ryff and Keyes, 1995; Keyes et al., 2002; Fisher, 2010). Several authors mention the importance of embracing both approaches in defining wellbeing, especially when testing the happy-productive worker thesis (e.g., Peiró et al., 2019, 2021). However, such definitions are rare in the literature, not to mention that prevailing conceptualizations of wellbeing fail to take into account the workplace context (Gutiérrez et al., 2020).

As one of the exceptions, Gilbert et al. (2011) and Gilbert and Malo (2017) propose a conceptualization of wellbeing framed within the organizational context and incorporate the eudemonic and hedonic approaches. According to those authors, psychological wellbeing at work is defined as a psychological state that includes cognitions and affects derived from the positive rapport to self, others, and the work environment. Positive rapport to oneself refers to serenity and being in a good mood, as well as feeling energetic and emotionally balanced at work. Positive rapport to others is about the pleasure that the individual experiences in his or her relationships at work, the feeling of being appreciated by others, and the perception of being able to remain oneself with one’s professional entourage. Finally, positive rapport to the work environment is defined by the feeling of being stimulated by one’s work, the desire to undertake projects, and the pursuit of goals in the work context (Gilbert et al., 2011; Gilbert and Malo, 2017). Taken together, these three dimensions describe a positive subjective experience in the workplace.

Often mistaken for job satisfaction or work engagement, psychological wellbeing at work differs in several ways (Gilbert and Malo, 2017). As mostly conceptualized in studies of the happy and productive worker thesis (Peiró et al., 2019), job satisfaction represents workers’ overall evaluation of their job. Compared to psychological wellbeing at work, which is composed of both affect and cognition (Gilbert et al., 2011; Gilbert and Malo, 2017), job satisfaction is more cognitive in nature and corresponds to workers’ overall experience.

Work engagement is defined as a positive state of accomplishment at work composed of vigor, dedication, and absorption (Schaufeli et al., 2002; Schaufeli and Bakker, 2004). Vigor can be seen as a significant amount of energy, a willingness to invest effort in work, and perseverance despite obstacles encountered. Dedication represents individuals’ enthusiasm, sense of purpose, and willingness to take on challenges. Absorption is characterized by a large concentration and deep immersion in one’s work, as well as a feeling that time is passing quickly and difficulty in detaching oneself from the tasks being carried out (Schaufeli et al., 2002). Whilst work engagement includes cognitive and affective components, as does psychological wellbeing at work, this positive subjective experience represents a more intense state. Moreover, according to conservation of resources theory, work engagement would be the result of a long process of accumulation of resources (Gorgievski and Hobfoll, 2008). Consequently, a considerable amount of resources seems to be required before reaching such a state. Since psychological wellbeing at work is not the result of such a lengthy process of resource accumulation, it would be more directly related to intrapersonal antecedents (Gilbert and Malo, 2017).

Considering these distinctions, the added value of studying psychological wellbeing at work rather than related variables can be seen in the nature of the construct, its proximity to different antecedents, as well as its work-specific conceptualization. In response to scholars’ invitation, namely Peiró et al. (2019), Gutiérrez et al. (2020), and Sender et al. (2021), self-determination theory (Deci and Ryan, 1985; Ryan and Deci, 2017) will serve as theoretical support for exploring intrapersonal antecedents of psychological wellbeing at work.

### 2.2 Self-determination and basic psychological needs

Self-determination theory (Deci and Ryan, 1985; Ryan and Deci, 2017) is one of the most influential theories in psychology regarding motivation (Hagger and Hamilton, 2021). At its core, the theory explains the inherent growth tendencies and the will to act according to free choice (Deci and Ryan, 2000; Ryan and Deci, 2017). This natural tendency reflects the central concept of self-determination, which refers to autonomous functioning, meaning that individuals would seek to undertake activities congruent with their self and regulate their behavior voluntarily and without coercion. According to Ryan and Deci (2017), basic psychological needs would be essential nutrients for such functioning and wellbeing.

Basic psychological needs theory (Ryan and Deci, 2017), one of six mini-theories included in self-determination theory, states that the need for autonomy, competence, and relatedness must be satisfied for self-determined functioning. Greater basic psychological need satisfaction would enable people to make choices consistent with their values and interests and engage in behaviors promoting wellbeing (Deci and Ryan, 2008; Ryan and Deci, 2017). For Ryan and Deci (2017), basic psychological need satisfaction can be examined in either general or context-specific terms, depending on the subject of study. Since the happy-productive worker thesis is embedded in the organizational context, we build on a work-specific conceptualization. Specifically, autonomy need satisfaction at work corresponds to the individuals’ perception of being in a workplace where they can exercise sufficient decisional, emotional, and behavioral latitude (Brien, 2011; Brien et al., 2012). Competence need satisfaction at work refers to the individuals’ perception of feeling up to the challenges they face in their job (Brien, 2011; Brien et al., 2012). Relatedness need satisfaction at work relates to the individuals’ sense of creating and having meaningful, reciprocal interpersonal connections within the professional circle (Brien, 2011; Brien et al., 2012).

Given the importance of basic psychological need satisfaction for self-determination, Ryan and Deci (2017) make several propositions for where the essential nutrients come from. In particular, they advance that social contexts offering autonomy support may facilitate basic psychological need satisfaction. For instance, autonomy-supportive practices include providing choices, positive feedback and showing consideration for others. Aside from social-contextual factors, the authors also argue that need satisfaction depends on person-specific variables. In this respect, they propose that mindfulness, an awareness of oneself and the context, serves as a foundation for self-determination and need satisfaction. Drawing upon these arguments, we extend the literature on happiness and performance by suggesting that mindfulness is an intrapersonal antecedent that could help people decide how to behave consistently with basic psychological need satisfaction at work. The reasoning behind our suggestion is developed below.

### 2.3 Mindfulness as an antecedent of basic psychological need satisfaction, wellbeing, and performance

As previously mentioned, Ryan and Deci (2017) state that most individuals strive to make decisions consistent with what they find important, relevant, meaningful, and in their best interest. In order to do so, they may process and evaluate events, which would help them make choices. Defined as an open and receptive awareness of what is happening both internally and externally in the present moment (Brown and Ryan, 2003), mindfulness allows individuals to access information from internal (e.g., needs, values, feelings) and external (e.g., social environment) sources.

Mindfulness is conceptualized as either a state or a trait. While the construct as a state represents temporary or transient occurrences of attention and awareness, trait mindfulness refers to individuals’ natural tendencies to be aware and to sustain attention (Brown and Ryan, 2003). Hence, trait mindfulness is more enduring than transient states, which may vary significantly over time (Brown and Ryan, 2003). Operationalizing mindfulness as a trait involves considering one’s natural inclination toward being mindful (Brown and Ryan, 2003; Mesmer-Magnus et al., 2017; Sala et al., 2020) without requiring a specific event or experience such as meditation (Brown and Ryan, 2003; Carpenter et al., 2019). Since studies on the happy-productive worker thesis have neglected individuals’ positive predispositions, exploring mindfulness as a dispositional tendency may advance research with a slightly broader view of intrapersonal antecedents.

In keeping with the self-determination theory’s formal propositions (Ryan and Deci, 2017), mindfulness would offer insight into one’s surroundings, as it entails openly attending to inner and outer experiences. Ryan and Deci (2017) propose that greater awareness of what’s going on inside and outside oneself may increase the possibility of voluntarily choosing and adopting behaviors. For the authors, the availability of various options would provide the autonomy necessary to satisfy needs for autonomy, competence, and relatedness, thereby potentially promoting self-determined functioning. When engaged in activities supporting basic psychological need satisfaction, individuals would likely demonstrate greater energy and vigor, possibly leading to enhanced wellbeing and more effective performance before, during, and after completing tasks, regardless of context (Ryan and Deci, 2017).

### 2.4 Proposed model

Building upon the theoretical propositions above, we argue that trait mindfulness would be indirectly linked to task performance via a sequential mediating effect of basic psychological need satisfaction and psychological wellbeing at work. First, trait mindfulness would predispose people to openly pay attention to their internal and external signals related to their needs, values, and interests at work, providing them with valuable information to guide their actions. The higher one’s dispositional tendency toward mindfulness, the more likely one would notice cues about the extent to which they have decisional latitude, feel able to meet professional challenges, and enjoy meaningful relationships in their work context. Drawing on the underlying logic of self-determination theory (Ryan and Deci, 2017), we posit that having access to such information might motivate people to select and engage in behaviors conducive to satisfying their needs for autonomy, competence, and affiliation at work. Supporting these ideas, prior research shows that trait mindfulness is related to autonomy need satisfaction (0.34 ≤ *r* ≤ 0.37; Brown and Ryan, 2003; *r* = 0.36, 95% CI = 0.31, 0.41; Van den Broeck et al., 2016), competence need satisfaction (0.39 ≤ *r* ≤ 0.68; Brown and Ryan, 2003; *r* = 0.39, 95% CI = 0.34, 0.43; Van den Broeck et al., 2016) and relatedness need satisfaction (0.28 ≤ *r* ≤ 0.31; Brown and Ryan, 2003; *r* = 0.28, 95% CI = 0.23, 0.33; Van den Broeck et al., 2016) in various work contexts (Van den Broeck et al., 2016), as well as adult and student samples (Brown and Ryan, 2003).

Second, we propose that the three need satisfactions at work would mediate the link between trait mindfulness and psychological wellbeing at work. Since people with a high level of trait mindfulness might be inclined to undertake actions consistent with their basic psychological needs in the organizational setting, they would thus be in favorable conditions for being authentically themselves. Accordingly, individuals are more likely to be in a good mood, stimulated by their job, remain themselves, and feel appreciated by their workplace’s entourage when they have decisional and behavioral latitude, the support needed to overcome job challenges, and enjoy reciprocal interpersonal relationships at work. Echoing our arguments, Chang et al. (2015) found, in a sample of undergraduate students, an indirect effect of trait mindfulness on eudemonic wellbeing at work via basic psychological need satisfaction measured as a global construct (β = 0.61, *p* < 0.001, 95% CI = 0.15, 0.29). They also reported the mediation effect of basic psychological need satisfaction linking trait mindfulness and positive affects (β = 0.34, *p* < 0.001, 95% CI = 0.08, 0.20), life satisfaction (β = 0.41, *p* < 0.001; 95% CI = 0.15, 0.41), and subjective wellbeing (i.e., hedonic wellbeing; Chang et al., 2015) measured as a composite score (β = 0.45, *p* < 0.001; 95% CI = 0.12, 0.23). Mesmer-Magnus et al.’ (2017) meta-analysis provides additional support for our conceptual proposition. Based on 270 studies conducted among adults from non-clinical samples, the results indicated a positive association between trait mindfulness and several wellbeing operationalizations (e.g., job satisfaction: ρ = 0.29, 95% CI = 0.23, 0.36, *k* = 18, *N* = 2,642).

While the proposition outlined encompasses a broader mechanism than what our model will specifically assess, including a set of variables (e.g., undertaking actions consistent with basic psychological need satisfaction at work) that are part of the motivational process described in self-determination theory (Deci and Ryan, 1985; Ryan and Deci, 2017), the empirical support presented above leads us to propose the following explanatory hypothesis:

H1: Trait mindfulness will be indirectly positively related to psychological wellbeing at work through the satisfaction of the basic psychological needs for (a) autonomy, (b) competence, and (c) relatedness at work.

Third, our last proposition is that trait mindfulness would be indirectly positively related to task performance via the satisfaction of the basic psychological needs for autonomy, competence, and relatedness at work, and through psychological wellbeing at work. Following our reasoning above derived from self-determination theory, trait mindfulness would help workers attune to and respond to their needs satisfactorily, thus offering essential nutrients for the self that would enhance psychological wellbeing at work. Because an integrated self and wellbeing would come with energy available for chosen behaviors (Ryan and Deci, 2017), psychological wellbeing at work may help people maintain a positive attitude toward activities like task performance. Although there is no direct evidence of these specific effects, several studies reported the mediating role of the basic psychological need satisfaction regarding the relationship between various operationalizations of wellbeing and performance. For example, Slemp et al. (2018) tested a meta-analytic path analysis of the link between leader autonomy support and positive work outcomes (i.e., general wellbeing, work engagement, job satisfaction, and positive work behavior) through both satisfaction of the basic psychological needs for autonomy, competence, and relatedness, and autonomous work motivation. The results showed that the model fits the data well [χ2(17) = 242.644, CFI = 0.977, TLI = 0.952, RMSEA = 0.048, CI = 0.043, 0.054, SRMR = 0.093, *N* = 5,667]. Providing additional support for our proposed model, van Wingerden et al. (2018) tested a sequential mediation linking job resources to task performance through basic psychological need satisfaction and work engagement. They collected data from 1,188 dyads of Dutch students and their internship supervisors in various occupational sectors. They found that interns’ basic psychological need satisfaction mediates the relationship between their job resources and work engagement (estimate = 0.706, *p* < 0.001; B-CCI [0.655, 0.753]), and that interns’ basic need satisfaction and work engagement mediate the relationship between their job resources and task performance (estimate = 0.297, *p* < 0.001, B-CCI [0.250, 0.342]). Furthermore, results revealed the indirect effect of basic psychological need satisfaction on task performance via work engagement (estimate = 0.344, *p* < 0.001, B-CCI [0.296, 0.391]; van Wingerden et al., 2018).

Since these papers mainly examined the mediation effect of basic psychological need satisfaction using an overall score, broader research is required to establish the singular contribution of each psychological need to the happy-productive worker thesis. On the other hand, the variety of indicators and contexts taken into account in the work above offer initial evidence supporting our conceptual model. We therefore propose a second hypothesis:

H2: Trait mindfulness will be indirectly positively related to task performance through the satisfaction of the basic psychological needs (a) autonomy, (b) competence, and (c) relatedness at work, and (d) psychological wellbeing at work.

## 3 Materials and methods

### 3.1 Participants and procedure

The research team received approval from the Research Ethics Committee of Université de Sherbrooke prior to collecting data for this study. Considering the exploratory scope of the project, we relied on a cross-sectional design and used a convenient snowball sampling method. Adopting equity, diversity, and inclusion practices (e.g., inclusive posting, self-identification questionnaire), we sent invitations via email and two social networks, Facebook and LinkedIn. Participation in the study was open to workers and organizations in Quebec, a Canadian province where French is the official language. Before accessing the questionnaire, participants had to read and agree to the terms of the research project as outlined in the consent form. On a voluntary basis, they anonymously completed an approximately 15-minute online survey, securely hosted in Canada on the SimpleSurvey platform.

Of the 296 French-speaking Canadian workers registered for our study, 283 provided usable data. In the final sample, 68.90% of participants identified as women, and the age ranged from 18 to 62 (*M* = 30.07, *SD* = 9.88). They were employed in various industries, including education, healthcare, finance, retail, and accommodation and food services. They were mostly employees (83.00%) and held a bachelor’s degree (32.90%). The average job tenure ranged from 0 to 42 years (*M* = 4.87, *SD* = 5.78), and the average organizational tenure ranged from 0 to 31 years (*M* = 4.54, *SD* = 5.67). Eligibility criteria for participation included being workers, 18 years or older, and sufficiently fluent in French to fill in the survey.

### 3.2 Measures

All measures were administered in French. Unless stated otherwise, participants were asked to respond to each item using a 7-point scale (from 1 = *strongly disagree* to 7 = *strongly agree*).

#### 3.2.1 Trait mindfulness

Trait mindfulness was measured using the short version of the Mindful Attention and Awareness Scale (MAAS; Brown and Ryan, 2003), i.e., the MAAS-Short (Höfling et al., 2011). For use purposes in this study, we translated the MAAS-Short measure into French following the back translation method published by Brislin (1970, 1986). We then conducted a confirmatory factor analysis (CFA) to examine the factor structure of this French version. Results showed wording effects attributed to the subtleties of the French language. This led us to remove four items [identified in Höfling et al. (2011) as maas10(-), maas11(-), maas14(-), maas11(+)] due to their grammatical redundancy (i.e., use of the verb “faire/doing” in all four items). Based on modification indices, we also covariated the residual errors of maas3(-) (i.e., reverse item) with mass3(+), and mass10(+) with mass14(+) (i.e., use of the verb “faire/doing” in all two items) to ensure having a manageable set of items that match and represent the construct definition. After this step, a new CFA was performed, and the results indicated that the model fit the data well, χ^2^(7) = 12.812, *p* < 0.05, χ^2^/*df* = 1.830, CFI = 0.988, TLI = 0.974, RMSEA = 0.055, 90% CI = 0.000, 0.101. Accordingly, we retained this version of 6-item scale (e.g., “Doing jobs or tasks with awareness”) for subsequent analyses. The instrument has a good internal consistency (α = 0.86), consistent with the findings for the original scale (MAAS: α = 0.87; Brown and Ryan, 2003) and the shortened one (MAAS-Short: α = 0.88; Höfling et al., 2011).

#### 3.2.2 Psychological wellbeing at work

The short and validated French version of the Psychological wellbeing at Work Scale (Gilbert and Malo, 2017) by Gilbert et al. (2011) was used to measure psychological wellbeing at work. It consists of nine items assessing the three construct’s dimensions, which are positive rapport to oneself (e.g., “Lately, in my job, I feel energetic”), positive rapport to others (e.g., “Lately, in my job, I remain myself with anyone”), and positive rapport to the work environment (e.g., “Lately, in my job, I feel like undertaking many things”). Each dimension is composed of three items rated on a 7-point scale (from 1 = *never* to 7 = *always*). The instrument holds a good internal consistency (α = 0.87), in line with the authors’ results (α = 0.86; Gilbert and Malo, 2017). In the current study, the model displays a good fit to the data, χ^2^(24) = 48.780, *p* < 0.005, χ^2^/*df* = 2.032, CFI = 0.979, TLI = 0.968, RMSEA = 0.061, 90% CI = 0.036, 0.085.

#### 3.2.3 Basic needs satisfaction at work

Participants completed the 12-item French measure from the Basic Psychological Needs at Work Scale (Brien et al., 2012). The instrument consists of three subscales of four items each to assess the satisfaction of basic psychological needs for autonomy (e.g., “My work allows me to make decisions”), competence (e.g., “I feel competent at work”), and relatedness at work (e.g., “When I am with the people of my work environment, I feel as though I can trust them”). The internal consistency is good (α_*autonomy*_ = 0.87; α_*competence*_ = 0.90; α_*relatedness*_ = 0.89), which is consistent with the work of Brien et al. (2012; 0.82 ≤ α_*autonomy*_ ≤ 0.87; 0.86 ≤ α_*competence*_ ≤ 0.92; 0.86 ≤ α_*relatedness*_ ≤ 0.92). Based on the CFA run for the current study, the fit statistics for this model is acceptable, χ^2^(54) = 163.743, *p* < 0.001, χ^2^/*df* = 3.032, CFI = 0.948, TLI = 0.937, RMSEA = 0.085, 95% CI = 0.070, 0.100.

#### 3.2.4 Task performance

We measured task performance with a French-validated version (Paiement et al., 2018) of Williams and Anderson’s (1991) scale. This adapted version includes six items (e.g., “Meets formal performance requirements of the job”), two of which are reversed (item 5 and item 6). Given the salient wording effect in French reversed items, the residual errors of item 5 and item 6 are allowed to covary (Paiement et al., 2018). The internal consistency is good (α = 0.82), in line with the results from Williams and Anderson (1991; α = 0.91). We performed a CFA and results show that the model fits the data well, χ^2^(8) = 4.587, *p* < 0.001, χ^2^/*df* = 0.573, CFI = 1.000, TLI = 1.022^1^, RMSEA = 0.000, 90% CI = 0.000, 0.045.

#### 3.2.5 Sociodemographic information

Participants provided demographic information, including gender, age, education, job and organizational tenure, and position occupied. Considering the importance of these variables for the happy-productive worker thesis (e.g., Harari et al., 2017; Petö and Reizer, 2021; Previtali and Spedale, 2021), we controlled for their potential effects in statistical analyses.

## 4 Results

### 4.1 Preliminary analyses

Following the recommendations of Tabachnick and Fidell (2019), preliminary analyses were performed before conducting the main analyses. Descriptive statistics (means and standard deviations) and correlations between the variables can be found in Table 1. The results show that all bivariate relationships are significant between the variables of interest.

**TABLE 1:** Descriptive statistics and correlations (*N* = 283).

Variables	*M*	*SD*	1	2	3	4	5	6
1. Trait mindfulness	5.131	0.922	(0.86)					
2. Satisfaction of autonomy need at work	5.679	1.078	0.212**	(0.87)				
3. Satisfaction of competence need at work	6.084	0.807	0.419**	0.347**	(0.90)			
4. Satisfaction relatedness need at work	5.242	0.675	0.419**	0.320**	0.388**	(0.89)		
5. Psychological wellbeing at work	5.421	0.828	0.370**	0.203**	0.569**	0.353**	(0.87)	
6. Task performance	6.074	0.675	0.452**	0.452**	0.521**	0.528**	0.415**	(0.82)

**p < 0.01.

#### 4.1.1 Missing data and basic premises

First, data entry was verified using response frequencies for each statement. The initial sample comprised 296 participants, 286 of whom provided complete answers. Based on the data screening, one participant was removed from the sample because the criterion of being age 18 years old or older was not met. Two other participants were removed because of unlikely response patterns (i.e., the same score was given to each statement on multiple scales). We therefore carried out subsequent analyses based on a total of 283 participants. The frequency analysis also revealed 21 missing data randomly distributed across the responses of different participants. Considering that the percentage of missing data represented less than 5% of the participants’ total responses and appeared to be randomly distributed, we replaced them by the means (Tabachnick and Fidell, 2019).

Second, the assumption of sample size is met since the sample consists of more than 200 participants (*N* = 283), and there are at least five participants for each model parameter to be estimated (Kline, 2016). Next, the absence of a dimension with an “index condition” higher than 30 indicates that multicollinearity and singularity are adequate (Belsley et al., 1980). In addition, there is no correlation exceeding 0.90, and the value of the tolerance index is greater than 0.10 while the value of VIF (variance inflation factor) is less than 10.00, which supports the absence of multicollinearity (Tabachnick and Fidell, 2019). Then, observation of the scatterplot of the standardized residuals revealed no irregularity, with respect to the homoscedasticity assumption. For univariate (i.e., 3.29 < Z scores < 3.29; Field, 2017) and multivariate (i.e., Cook’s distance < 1; Field, 2017) extreme values, no participants were removed. Linearity between variables was verified by observing the standardized residual plot (Tabachnick and Fidell, 2019). Finally, the normality of the distribution was examined for all variables, although the bootstrapping procedure used in this study is robust enough to the normality assumption (Hayes, 2017). The results show that all variables are normally distributed as they have skewness indices that range from −3 to 3 and kurtosis indices that range from −10 to 10 (Kline, 2016).

#### 4.1.2 Control variables

In line with the writings of Becker (2005), we tested the demographic variables’ effect on the two key variables of the happy-productive worker thesis (i.e., psychological wellbeing at work and task performance) to verify the relevance of including those variables in the main analyses. To this end, correlation analyses for continuous variables and variance analyses for categorical variables were performed. No significant effect was found for the demographic variables (i.e., gender, age, education, job and organizational tenure, and position occupied). Therefore, no control variables have been included in the subsequent analyses.

### 4.2 Main analyses

Analyses were performed using structural equation modeling employing the maximum likelihood estimation in Mplus 8.0. The following indices were used to assess the goodness of fit of the different models tested: the ratio of the chi-square divided by its degrees of freedom (χ^2^/*df*), the Comparative Fit Index (CFI), the Tucker-Lewis Index (TLI), and the Root Mean Square Error of Approximation (RMSEA), including the 90% confidence intervals. Referring to the recommendations of Marsh and Hocevar (1985), a χ^2^/*df* ratio of 2:1 to 5:1 is considered an acceptable fit to the data. CFI and TLI values greater than 0.90 (Hu and Bentler, 1995) represent an indicator of a good model fit to the data, whereas values reaching or exceeding 0.95 are considered an excellent model fit (Hu and Bentler, 1999). Similarly, an RMSEA value below 0.08 indicates a good model fit, while a value below 0.05 indicates an excellent fit to the data (Hu and Bentler, 1999), including 90% confidence intervals (Kline, 2011; Byrne, 2013). For comparison purposes between different examined models, we used the Satorra–Bentler χ^2^ difference test incorporating the Maximum-Likelihood Restricted scaled correction factors (Satorra and Bentler, 2001).

#### 4.2.1 Measurement models

Since measurement models specify the relationships between observed variables and latent variances, we compared five measurement models to ensure measuring the six constructs related to the theoretical model. The first tested model represents our proposed model. Parcels were created to limit the number of parameters of the model under study, thereby following the recommendations of Little et al. (2002). Global scores of the three dimensions of psychological wellbeing at work were used to create those parcels. Consequently, the first model comprises six factors, which are the latent variables of the study, and includes 27 observed variables. The results show that the model fits the data well, χ^2^(306) = 530.542, *p* < 0.001, χ^2^/*df* = 1.734, CFI = 0.932, TLI = 0.922, RMSEA = 0.051, 90% CI = 0.044, 0.058. In line with Cortina et al.’s (2020) recommendations, we conducted a comparative CFA to rule out alternatives to the previous model. Indeed, a second model with four factors was tested. Four alternative models were then tested, and results from those analyses show superior fit to the data for the first model than the alternative ones. Therefore, the first model composed of six factors is chosen to test the structural models. Table 2 details the results of these analyses.

**TABLE 2:** Comparison of measurement and structural models (*N* = 283).

Models	χ^2^	*df*	χ^2^*/df*	△χ^2^ (*df*)	CFI	TLI	RMSEA (CI 90%)
**Measurement Models**							
Proposed and Final Model	530.542***	306	1.734	–	0.932	0.922	0.051
(Factor 1: Trait Mindfulness; Factor 2: BNS-AW; Factor 3: BNS-CW; Factor 4: BNS-RW; Factor 5: PWBW; Factor 6: TP; baseline model for comparison with all next measurement models)							(0.044, 0.058)
4-Factor Model	1394.558***	315	4.427	611.454**	0.675	0.638	0.110
(Factor 1: Trait Mindfulness; Factor 2: BNS-W; Factor 3: PWBW; Factor 4: TP)				(9)			(0.104, 0.116)
3-Factor Model	1589.606***	318	4.999	614.138**	0.617	0.578	0.119
(Factor 1: Trait Mindfulness and BNS-W; Factor 2: PWBW; Factor 3: TP)				(12)			(0.113, 0.125)
2-Factor Model	1623.661***	320	5.074	646.778**	0.608	0.570	0.120
(Factor 1: Trait Mindfulness, BNS-W; and PWBW; Factor 2: TP)				(14)			(0.114, 0.126)
1-Factor Model	1823.03***	321	5.679	599.945**	0.548	0.506	0.129
(All items combined into a single factor)				(15)			(0.123, 0.134)
**Structural Models**							
Proposed Model	576.028***	311	1.852	–	0.920	0.910	0.055
(Baseline model for comparison with next model)							(0.048, 0.062)
Alternative Model 1	530.541***	306	1.734	46.865**	0.932	0.922	0.051
(Direct and indirect effect; baseline model for comparison with next model)				(5)			(0.044, 0.058)
Alternative Model 2 and Final Model	536.205***	309	1.735	5.754	0.932	0.922	0.051
(PWBW and TP as dependent variables and non-significant paths dropped; baseline model for comparison with next model)				(3)			(0.044, 0.058)
Alternative Model 3	640.807***	311	2.060	95.065**	0.901	0.888	0.061
(TP put before PWBW)				(2)			(0.054, 0.068)

PWBW, psychological wellbeing at work; BNS-AW, autonomy need satisfaction at work; BNS-CW, competence need satisfaction at work; BNS-RW, relatedness need satisfaction at work; BNS-W, basic need satisfaction at work. We point to the final models in bold characters in the table.

**p < 0.01.

***p < 0.001.

#### 4.2.2 Structural models

As structural models allow for the analysis of relationships between the studied constructs (Kline, 2016), four models were tested accordingly to the research hypotheses. The first model tested includes all research hypotheses. Specifically, it incorporates the indirect effect of trait mindfulness on task performance through the satisfaction of the basic psychological needs for autonomy, competence, and relatedness at work, and via psychological wellbeing at work. Results reflect that the model fit the data well, χ^2^(311) = 576.028, *p* < 0.001, χ^2^/*df* = 1.852, CFI = 0.920, TLI = 0.910, RMSEA = 0.55, 90% CI = 0.048, 0.062.

Following James et al.’s (2006) recommendation, we ran a second model including both direct and indirect effects for comparison with the previous model. Beyond this empirical justification, there is also theoretical reasoning behind the second model. According to the conservation of resources theory, individuals strive to maintain, protect, and develop resources, which may explain much of human behavior (Hobfoll et al., 2018). To acquire resources and address demands at work, people would have to invest their available ones. Those resources can include psychological characteristics (e.g., autonomy, competence, self-esteem), objects (e.g., housing, clothing), energy (e.g., time, knowledge), and conditions (e.g., job security, social support; Hobfoll, 1998). Applying these theoretical considerations to our context, we propose that the study’s variables may be considered available psychological resources in meeting job requirements, which include task performance. Therefore, we explored a second model combining direct and indirect effects. Despite the model indices fit the data being better than the previous ones, findings reveal that trait mindfulness, the satisfaction of the basic psychological needs for autonomy, and relatedness at work, as well as psychological wellbeing at work, are not significantly related to task performance.

Given the preceding results, we tested a third model where both psychological wellbeing at work and task performance are concurrently dependent variables at the same level, and all non-significant paths were removed. Compared to the previous alternative model, model indices fit do not increase, Satorra-Bentler △χ^2^ = 5.754, *df* = 3, *ns*, and all significant paths remain significant. Thereupon, we tested a fourth and last model to verify if the sequence of the variables presented in previous models is the most probable. Hence, we put task performance before psychological wellbeing at work. Specifically, the fourth model combines the indirect effect of trait mindfulness on psychological wellbeing at work via the satisfaction of the basic psychological needs for autonomy, competence, and relatedness at work, and through task performance. This model yields lower fit indices than previous ones, χ^2^(311) = 640.807, *p* < 0.001, χ^2^/*df* = 2.060, CFI = 0.901, TLI = 0.888, RMSEA = 0.061, 90% CI = 0.054, 0.068. Satorra-Bentler △χ^2^ = 95.065, *df* = 2, *p* < 0.01. Table 2 presents the results for all structural models analyzed. To conduct hypothesis testing, we built on James et al.’s (2006) work by selecting the most parsimonious, theory-aligned model. Therefore, we based our subsequent analyses on the third model.

#### 4.2.3 Verifying the hypotheses

Verification of research hypotheses was accomplished by conducting analyses employing the bootstrapping method from 1,000 samples and based on a 95% confidence interval (Shrout and Bolger, 2002; MacKinnon et al., 2004; Cheung and Lau, 2008). Confidence intervals were calculated using standardized regression coefficients to ensure that no sample included a value of zero, which would indicate no indirect effect. Furthermore, we used the percentage of variance represented by the squared association index (*R*^2^) following Ferguson’s (2016) criteria to test for effect size. According to these, a value of 0.04 represents a small effect, a value of 0.25 is considered a moderate effect, and a value of 0.64 represents a strong effect.

Results show that trait mindfulness is indirectly positively related to psychological wellbeing at work through the satisfaction of the basic psychological needs for autonomy (0.060, 95% CI = 0.012, 0.121), competence (0.145, 95% CI = 0.058, 0.239), and relatedness at work (0.129, 95% CI = 0.065, 0.204). Those findings provide support for Hypothesis 1a-c.

**FIGURE 1:**
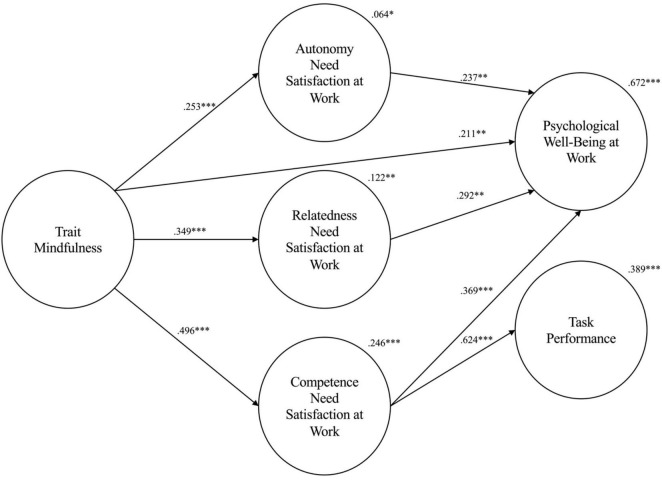
Final Model. *p < 0.05; **p < 0.01; ***p < 0.001.

Regarding Hypothesis 2a-d, results indicate that trait mindfulness is not significantly indirectly positively associated with task performance through the hypothesized sequential mediating effect of basic psychological need satisfaction and psychological wellbeing at work. Thus, Hypothesis 2a-d is not supported. We performed *post hoc* analyses based on our final model to examine the significant effects. Figure 1 shows the results of our analyses. Overall, the general model explained 67.2% of the variance in psychological wellbeing at work (i.e., strong effect) and 38.9% in task performance (i.e., moderate effect).

## 5 Discussion

This study sought to move beyond earlier work on the happy-productive worker thesis by testing a new pair of happy construct-productive criteria. Shedding light on the relationship between a work-specific conceptualization of wellbeing and task performance, our findings suggest that one does not necessarily come before the other. Rather than supporting the initially proposed sequential mediation, our work showcases two distinct mediation processes, one explaining psychological wellbeing at work and the other task performance. When prior research focuses mainly on the direction of the relationships between happiness and performance (Gutiérrez et al., 2020), our results pave the way for additional investigation, putting both constructs on an equal footing. Moreover, we expand their nomological network by taking into account factors outside the realm of dark propensities. Integrating past evidence with self-determination theory (Deci and Ryan, 1985; Ryan and Deci, 2017), we bring a fresh perspective by exploring the basic psychological need satisfaction alongside an intrapersonal antecedent that makes human beings the focus of their own experience. We found indirect effects of trait mindfulness on both wellbeing and performance through the differential contribution of the satisfaction of the need for autonomy, competence, and relatedness at work. Our findings open possibilities to investigate, both in theory and practice, the ongoing quest for the Holy Grail. The following paragraphs provide an overview of some of these opportunities.

### 5.1 Theoretical implications

By delving into the link between trait mindfulness and task performance through basic psychological need satisfaction and psychological wellbeing at work, the current research contributes in many ways to the scientific literature. First, our study heeds Peiró et al.’ (2019) call to build on new conceptualizations of happiness in the happy-productive worker thesis by underscoring the singularity of psychological wellbeing at work. With respect to Johns’ (2006; 2017) statement on the contribution of context to the explanation of organizational behavior, we thus mark an advance from past research by relying on a work-specific operationalization of wellbeing. Since the happy-productive worker thesis lies within the organizational settings, focusing on positive experiences and the basic psychological need satisfaction proper to this context, compared with context-free concepts, may offer a more accurate and practical depiction of what occurs in real-life workplaces.

Second, we respond to the invitation of Peiró et al. (2021) and Sender et al. (2021) by clarifying the role of trait mindfulness, which yet received little attention as an antecedent in the literature on the happy-productive worker thesis. Our findings support the first hypothesis – individuals who report being highly aware of their surroundings and inner thoughts experience greater satisfaction of their basic psychological needs for autonomy, competence, and relatedness, and psychological wellbeing at work. These results reflect the general patterns predicted by self-determination theory about the effect of mindfulness on basic psychological need satisfaction, as well as the effect of basic psychological need satisfaction on wellbeing (Ryan and Deci, 2017).

Our findings also add to a growing body of evidence linking mindfulness to orbiting wellbeing indicators, including job satisfaction (Mesmer-Magnus et al., 2017) and positive affect (Brown and Ryan, 2003). Additionally, the current research echoes results on the mediation effect of basic psychological need satisfaction between mindfulness and positive subjective experiences, such as eudemonic wellbeing (Chang et al., 2015), vitality (Chang et al., 2018) and life satisfaction (Chang et al., 2015, 2018). Whereas these studies involve athletes (Chang et al., 2018), undergraduate students (Brown and Ryan, 2003; Chang et al., 2015), adults from non-clinical samples (Mesmer-Magnus et al., 2017), and workers (Brown and Ryan, 2003), we turned our attention to a population almost absent from papers on the happy-productive worker thesis, namely French-speaking Canadian workers. In doing so, we broaden the scope of previous efforts by addressing Sender et al.’s (2021) call to look beyond USA borders for the greater good of the happy-productive worker thesis.

Third, our results reveal that contrary to our initial hypothesis, psychological wellbeing at work does not act as a mediating variable in the relationship between trait mindfulness and task performance. Instead of observing a sequential mediating effect, we found an indirect effect of trait mindfulness on task performance via the satisfaction of the basic psychological need for competence at work. Unexpectedly, autonomy and relatedness need satisfactions at work do not mediate the link between trait mindfulness and task performance. From a theoretical perspective, we suggest two main explanations for these significant and non-significant findings. On the one hand, our results remain consistent with self-determination theory (Ryan and Deci, 2017) even though the sequential mediation is not supported. Specifically, Ryan and Deci’s manifold theoretical propositions offer alternatives for positioning psychological wellbeing at work and task performance at the same level as dependent variables. As mentioned earlier, the authors argued that individuals engaged in activities promoting basic psychological need satisfaction will likely feel more energy and vigor. Such an energetic state would benefit both wellbeing and performance (Ryan and Deci, 2017). This particular part of the authors’ proposal offers a comprehensive view of the two constructs without enforcing a specific viewpoint on how they are related. Therefore, it allows us to anchor our work to interpret and use our results. Forthcoming research is needed to empirically test this preliminary interpretation by exploring how individuals’ actions to satisfy their psychological needs and energetic state operate with trait mindfulness to explain the above relationships.

On the other hand, the social cognitive theory (Bandura, 1986, 2001) provides insights that may explain the indirect effect of trait mindfulness on task performance via only one of the three need satisfactions at work. Bandura (2001) proposed that human functioning results from the interplay of intrapersonal, behavioral, and environmental factors and asserted that individuals are active agents able to deliberately influence their functioning and environment through their actions. The more confident they are in their capabilities to accomplish given attainments, the more likely they would be motivated to make the necessary efforts to persevere and produce behaviors accordingly. This idea of being able to perform desired attainments crosses over with the definition of the competence need satisfaction at work since it refers to individuals’ perception of feeling up to the challenges faced in their job. Following Bandura’s logic, we propose that the more workers would feel having what they need to meet the challenges in their work environment, the more they would feel able to carry out consistent actions, and therefore, the more they would engage in behaviors their job implies, i.e., task performance. Differently, autonomy and relatedness need satisfactions at work do not relate to a feeling of mastery and effectiveness in the organizational context. Indeed, autonomy need satisfaction at work reflects a feeling of exercising one’s own influence over one’s actions and choices, while relatedness need satisfaction at work focuses on the feeling of being accepted by one’s occupational surroundings. Based on these conceptual distinctions, we might suggest that the competence need satisfaction at work is conceptually more proximal to task performance than the other two constructs, thereby providing a stronger contribution in explaining the dependent variable’s variance. Consistent with this reasoning, our results indicate that the satisfaction of each three needs at work positively correlates with task performance. However, only the effect of competence need satisfaction at work remains significant after accounting for all three variables in the multiple regression model. Therefore, our study provides evidence in line with the findings of Hoxha and Çetin (2020), who report that only the basic psychological need for competence is significantly related to task performance when testing the effects of basic psychological need satisfaction among individuals working in public companies operating in post and telecommunication. Additional research is required to further disentangle the effects of trait mindfulness on task performance by delineating the unique contributions of both task performance self-efficacy and the satisfaction for basic psychological needs at work, with an emphasis on competence need satisfaction at work.

Finally, we advance research on the happy-productive worker thesis by investigating a new pair of wellbeing-performance constructs in a larger model which accounts for dispositional and motivational antecedents. As regards scientific and practical relevance, integrating trait mindfulness into the relationship between psychological wellbeing at work and task performance stands out against the general inclination toward more conventional personality traits (e.g., neuroticism). Given the long tradition in psychology of focusing on fixing what is wrong with individuals (e.g., Compton and Hoffman, 2020), we extend this perspective by dwelling on people’s active role in explaining their wellbeing and performance in the workplace. Aside from fulfilling requests made by several scholars (e.g., Gutiérrez et al., 2020; Sender et al., 2021), our study incites to rethink the link between those two indicators. Rather than continuing the age-old debate on the assumed directionality, we propose to build on current results by treating wellbeing and performance as two dependent variables possibly operating concurrently. This approach is consistent with the view of Judge et al. (2017), who acknowledge that “the concern with “human happiness” is as legitimate and socially important as the concern with efficiency” (p. 356), and the proposal by Ipsen et al. (2020) to move from viewing psychological health and performance as disconnected and begin to study them in tandem. Our recommendation also aligns with studies assessing both constructs in terms of outcomes (e.g., Slemp et al., 2018; Armand et al., 2021; Kadir and Broberg, 2021). Altogether, the above considerations point to the need for further research into the antecedents that can simultaneously foster wellbeing and performance in organizational settings.

### 5.2 Practical implications

From a practical perspective, our findings allow us to reassert the value of promoting work environments tailored to the needs of their members. For further food for thought in this direction, a meta-analysis of 72 studies of workers’ samples from Slemp et al. (2018) showed that leader autonomy support was positively associated with mindfulness, basic psychological need satisfaction, general wellbeing, hedonic wellbeing, eudemonic wellbeing, and performance. Examples of such autonomy-supportive practices from supervisors include encouraging and assisting their employees in doing the tasks, considering their perspectives, providing them opportunities for choice and feedback, and promoting initiative taking (Slemp et al., 2018).

Perhaps even more important, our results shed light on the promising role of trait mindfulness in people’s experience within the workplace. In one fell swoop, this intrapersonal antecedent would act as a leverage point to promote several important aspects of quality of life at work, namely basic psychological need satisfaction, psychological wellbeing at work, and task performance. To foster mindfulness, organizations are invited to focus on ways to help their employees get off autopilot and focus on the present moment. For example, listening to and legitimizing people’s needs (e.g., physiological and psychological; Toniolo-Barrios and Pitt, 2021) could be ways of actively engaging team members in spending time paying attention to their internal cues and adopting behaviors that enable them to meet their needs. Whereas such an approach requires effort, workload management represents a potential issue to be addressed. Indeed, excessive or contradictory demands can lead to overload (Oplatka, 2017; Hülsheger et al., 2018), hampering attempts to be aware of present-moment experiences (Hülsheger et al., 2018). To meet the challenge, organizations would benefit from support in setting up working conditions that keep workloads at a manageable pace and give opportunities for recovery. Allowing individuals the freedom to take brief breaks may be worthwhile to replenish their resources when they feel overwhelmed with work demands (Hülsheger et al., 2018).

Another way to help workers unplug the autopilot would be to take moments of digital disconnection (Syvertsen, 2020; Syvertsen and Enli, 2020). For instance, they could silence their phones when they need to focus 100% on their tasks. In doing so, they could be fully present and limit distractions from incoming notifications, such as calls and emails (Syvertsen, 2020; Syvertsen and Enli, 2020). In addition, given that individuals can serve as role models for their peers and positively influence organizational practices (e.g., Mohamed Osama and Gallagher, 2018; Boldureanu et al., 2020), organizations could introduce mechanisms to identify and recognize people who stand out for their capacity for mindfulness (Dust et al., 2022). Moreover, setting up office spaces dedicated to mindfulness practices and offering meditation workshops could promote trait mindfulness. Supporting this idea, an 8-week mindfulness intervention study finds that developing individuals’ state mindfulness with repeated meditation sessions leads to greater trait mindfulness and less psychological distress (Kiken et al., 2015). When participants reached a deeper state of mindfulness during meditation, they were likelier to adopt mindfulness attitudes and behaviors outside of meditation, such as within the workplace (Kiken et al., 2015).

### 5.3 Limitations and research agenda

Despite the contributions made by this study, our results must be interpreted with caution due to certain limitations. First, as the exploratory objective of this study was to examine relationships between variables of interest, we opted for a cross-sectional research design. While this design has the advantage of providing initial evidence before undertaking a more extensive, more time-and energy-intensive study, it does not allow us to draw conclusions about the causal relationships between the constructs examined. Building on those preliminary results, future work could address this limitation by relying on experimental research designs or further examine the proposed model by separating the independent, mediator, and dependent variables over time using a longitudinal design.

Second, although the snowball method of sampling had several advantages, such as reaching people quickly and a high proportion of the general population, as well as being a contemporary method (e.g., invitation through social networks), it is unfortunately not possible to calculate the actual response rate, as it is not possible to know how many people were reached by invitation. However, we were able to reach a population that has received very little attention in the literature on the happy-productive worker thesis. Nevertheless, further research should increase the generalizability of the conclusions drawn from the results since our sample comprises French-speaking Canadian workers and educated women. It would be beneficial to expand the sample to include a greater diversity of genders and cultures, as well as to vary the sampling methods by targeting various backgrounds for a better representation of the worker population. To do so, scholars are invited to broaden the survey population, for instance, by selecting managers and male-dominated jobs such as police officers, engineers, and firefighters.

Third, using a self-reported measure for task performance should be considered when interpreting the results, as participants may have overestimated their responses (Gude et al., 2018). Therefore, additional efforts could be made to use multiple sources of information to broaden the explication of the relationship between happiness and performance. By collecting managers’ perceptions of their employees’ task performance, we could test our proposed model from a new perspective, thereby enriching the understanding of the link between trait mindfulness, psychological wellbeing, and task performance.

Finally, although the present work revisits the happy-productive worker thesis by including six variables, additional factors warrant further scholarly attention in order to more robustly and thoroughly examine the process outlined in our theoretical model. To do so, future research could explore individuals’ motivational processes leading to the satisfaction of basic psychological needs while integrating the proposed yet untested mediators, such as decision latitude, feedback seeking, and the creation of meaningful relationships. A final recommendation would be for future inquiries to account for a range of happiness operationalizations simultaneously, including positive subjective experiences (e.g., job satisfaction, work engagement) and negative ones (e.g., burnout).

## 6 Conclusion

Rather than looking at psychological wellbeing at work as a variable coming before task performance, our study paves the way for further efforts in investigating both constructs in tandem. Furthermore, this article outlines an emergent intrapersonal antecedent of the essential nutrients for individuals’ functioning according to self-determination theory (Deci and Ryan, 1985; Ryan and Deci, 2017), thereby contributing to the literature on the happy-productive worker thesis. Regarding potential implications, we call on the scientific and practical community to explore how employees can disengage from their autopilot and be more connected to their needs. In the reality of labor shortages, work overload, psychological distress among employees, and more, paying attention to mindfulness may seem challenging for organizations and workers. More research is thus needed to explore how people can meet this challenge despite their context. By embracing an approach rooted in mindfulness, subsequent work will take the next step in highlighting the role that individuals can play in their own work experience and behaviors.

## Data availability statement

The datasets presented in this article are not readily available because access to the data is restricted to protect confidential information. Requests to access the datasets should be directed to MM, marie.malo@usherbrooke.ca.

## Ethics statement

The studies involving humans were approved by the Comité d’Éthique de la Recherche; Lettres et Sciences Humaines. The studies were conducted in accordance with the local legislation and institutional requirements. The participants provided their written informed consent to participate in this study.

## Author contributions

RG: Formal analysis, Writing—original draft, Writing—review and editing, Conceptualization. MM: Conceptualization, Formal analysis, Investigation, Methodology, Project administration, Supervision, Validation, Writing—review and editing. M-HG: Conceptualization, Investigation, Methodology, Project administration, Supervision, Validation, Writing—review and editing.

## Footnotes

1 According to Muthén and Muthén (2017), it is common for the TLI value to exceed 1 when the sample size is relatively small.

## References

[B1] Armand M. A. Biassoni F. Corrias A. (2021). Sleep, well-being and academic performance: A study in a Singapore residential college. *Front. Psychol.* 12:672238. 10.3389/fpsyg.2021.672238 34135831 PMC8200680

[B2] Arshad M. A. Arshad D. Zakaria N. (2023). Mediating role of wellbeing among organizational virtuousness, emotional intelligence and job performance in post-pandemic COVID-19. *Front. Psychol.* 14:1105895. 10.3389/fpsyg.2023.1105895 36777235 PMC9911677

[B3] Ayala Y. Peiró Silla J. M. Tordera N. Lorente L. Yeves J. (2017). Job satisfaction and innovative performance in young Spanish employees: Testing new patterns in the happy-productive worker thesis—a discriminant study. *J. Happ. Stud.* 18 1377–1401. 10.1007/s10902-016-9778-1

[B4] Bandura A. (1986). *Social foundations of thought and action: A social cognitive theory.* Hoboken, NJ: Prentice-Hall.

[B5] Bandura A. (2001). Social cognitive theory: An agentic perspective. *Annu. Rev. Psychol.* 52 1–26. 10.1146/annurev.psych.52.1.1 11148297

[B6] Barnett P. A. Mackintosh B. Sapountzis A. (2009). Paying attention to anxiety: Attentional control and anxiety in children. *J. Anxiety Disord.* 23, 222–230.

[B7] Becker T. E. (2005). Potential problems in the statistical control of variables in organizational research: A qualitative analysis with recommendations. *Organ. Res. Methods* 8 274–289. 10.1177/1094428105278021

[B8] Belsley D. A. Kuh E. Welsch R. E. (1980). *Regression diagnostics: Identifying influence data and source of collinearity.* Hoboken, NJ: Wiley.

[B9] Boldureanu G. Ionescu A. M. Bercu A.-M. Bedrule-Grigoruţă M. Boldureanu D. (2020). Entrepreneurship education through successful entrepreneurial models in higher education institutions. *Sustainability* 12:1267. 10.3390/su12031267

[B10] Brien M. (2011). *La satisfaction des trois besoins fondamentaux peut-elle contribuer à la performance? L’apport de la santé psychologique*. Ph.D Thesis. Montreal, QC: Université de Montréal.

[B11] Brien M. Forest J. Mageau G. A. Boudrias J.-S. Desrumaux P. Brunet L. (2012). The basic psychological needs at work scale: Measurement invariance between Canada and France: Basic psychological needs at work scale. *Appl. Psychol. Health WellBeing* 4 167–187. 10.1111/j.1758-0854.2012.01067.x 26286976

[B12] Brislin R. W. (1970). Back-translation for cross-cultural research. *J. Cross Cult. Psychol.* 1 185–216. 10.1177/13591045700010030

[B13] Brislin R. W. (1986). “The wording and translation of research instruments,” in *Field methods in cross-cultural research*, eds Lonner W. J. Berry J. W. (Thousand Oaks, CA: Sage Publications, Inc), 137–164.

[B14] Brown K. W. Ryan R. M. (2003). The benefits of being present: Mindfulness and its role in psychological well-being. *J. Pers. Soc. Psychol.* 84 822–848. 10.1037/0022-3514.84.4.822 12703651

[B15] Byrne B. M. (2013). *Structural equation modeling with LISREL, PRELIS, and SIMPLIS: Basic concepts, applications, and programming.* London: Psychology Press.

[B16] Carpenter J. K. Conroy K. Gomez A. F. Curren L. C. Hofmann S. G. (2019). The relationship between trait mindfulness and affective symptoms: A meta-analysis of the Five Facet Mindfulness Questionnaire (FFMQ). *Clin. Psychol. Rev.* 74:101785. 10.1016/j.cpr.2019.101785 31751877 PMC6878205

[B17] Chang J.-H. Huang C.-L. Lin Y.-C. (2015). Mindfulness, basic psychological needs fulfillment, and well-being. *J. Happ. Stud.* 16 1149–1162. 10.1007/s10902-014-9551-2

[B18] Chang W. H. Chang J.-H. Chen L. H. (2018). Mindfulness enhances change in athletes’ well-being: The mediating role of basic psychological needs fulfillment. *Mindfulness* 9 815–823. 10.1007/s12671-017-0821-z

[B19] Cheung G. W. Lau R. S. (2008). Testing mediation and suppression effects of latent variables: Bootstrapping with structural equation models. *Organ. Res. Methods* 11 296–325. 10.1177/1094428107300343

[B20] Compton W. C. Hoffman E. (2020). “An introduction to positive psychology,” in *Positive psychology: The science of happiness and flourishing*, eds Compton W. C. Hoffman E. (Thousand Oaks, CA: Sage Publications, Inc), 1–28.

[B21] Cortina J. M. Sheng Z. Keener S. K. Keeler K. R. Grubb L. K. Schmitt N. (2020). From Alpha to Omega and Beyond! A look at the past, present, and (Possible) future of psychometric soundness in the journal of applied psychology. *J. Appl. Psychol.* 105 1351–1381. 10.1037/apl0000815 32772525

[B22] Cropanzano R. Wright T. A. (1999). A 5-year study of change in the relationship between well-being and job performance. *Consult. Psychol. J. Pract. Res.* 51 252–265. 10.1037/1061-4087.51.4.252

[B23] Cropanzano R. Wright T. A. (2001). When a “happy” worker is really a “productive” worker: A review and further refinement of the happy-productive worker thesis. *Consult. Psychol. J. Pract. Res.* 53 182–199. 10.1037/1061-4087.53.3.182

[B24] Deci E. L. Ryan R. M. (1985). *Intrinsic motivation and self-determination in human behavior.* New York, NY: Plenum.

[B25] Deci E. L. Ryan R. M. (2000). The “what” and “why” of goal pursuits: Human needs and the self-determination of behavior. *Psychol. Inq.* 11 227–268. 10.1207/S15327965PLI1104_01

[B26] Deci E. L. Ryan R. M. (2008). Facilitating optimal motivation and psychological well- being across life’s domains. *Can. Psychol.* 49 14–23. 10.1037/0708-5591.49.1.14

[B27] Diener E. (2000). Subjective well-being: The science of happiness and a proposal for a national index. *Am. Psychol.* 55 34–43. 10.1037/0003-066X.55.1.34 11392863

[B28] Dust S. B. Liu H. Wang S. Reina C. S. (2022). The effect of mindfulness and job demands on motivation and performance trajectories across the workweek: An entrainment theory perspective. *J. Appl. Psychol.* 107 221–239. 10.1037/apl0000887 33829831

[B29] Ferguson C. J. (2016). “An effect size primer: A guide for clinicians and researchers,” in *Methodological issues and strategies in clinical research*, ed. Kazdin A. E. (Washington, DC: American Psychological Association), 301–310. 10.1037/14805-020

[B30] Field A. (2017). *Discovering statistics using IBM SPSS statistics*, 5th Edn. Thousand Oaks, CA: Sage Publications Ltd.

[B31] Fisher C. D. (2010). Happiness at work. *Int. J. Manag. Rev.* 12 384–412. 10.1111/j.1468-2370.2009.00270.x

[B32] Fogaça N. Coelho Junior F. Paschoal T. Ferreira M. C. (2021). Relationship between job performance, well-being, justice, and organizational support: A multilevel perspective. *Rev. Admin. Mackenzie* 22 1–27. 10.1590/1678-6971/eRAMG210108

[B33] Ghasemy M. Gaskin J. E. Elwood J. A. (2023). Testing the “holy grail” of industrial psychology as a non-recursive bow pattern model in higher education using the PLSe2 method. *J. Appl. Res. High. Educ.* [Epub ahead of print] 10.1108/JARHE-10-2022-0333

[B34] Gilbert M.-H. Malo M. (2017). “Psychological health at work: A measurement scale validation [Communication],” in *Proceedings of the 25th congress of the European association of work and organisational psychology*, Dublin.

[B35] Gilbert M.-H. Dagenais-Desmarais V. Savoie A. (2011). Validation d’une mesure de santeì psychologique au travail. *Eur. Rev. Appl. Psychol.* 61 195–203. 10.1016/j.erap.2011.09.001

[B36] Gorgievski M. J. Hobfoll S. E. (2008). “Work can burn us out or fire us up: Conservation of resources in burnout and engagement,” in *Handbook of Stress and Burnout in Health Care*, ed. Halbesleben J. R. P. (Hauppauge, NY: Nova Science Publishers), 7–22.

[B37] Gu X. Obrenovic B. Fu W. (2023). Empirical study on social media exposure and fear as drivers of anxiety and depression during the COVID-19 pandemic. *Sustainability* 15:5312. 10.3390/su15065312

[B38] Gude W. T. Roos-Blom M.-J. van der Veer S. N. Dongelmans D. A. de Jonge E. Francis J. J. (2018). Health professionals’ perceptions about their clinical performance and the influence of audit and feedback on their intentions to improve practice: A theory-based study in Dutch intensive care units. *Implement. Sci.* 13:33. 10.1186/s13012-018-0727-8 29454393 PMC5816547

[B39] Gutiérrez O. I. Polo J. D. Zambrano M. J. Molina D. C. (2020). Meta-analysis and scientific mapping of well-being and job performance. *Spanish J. Psychol.* 23 1–22. 10.1017/SJP.2020.40 33107425

[B40] Hagger M. S. Hamilton K. (2021). General causality orientations in self-determination theory: Meta-analysis and test of a process model. *Eur. J. Pers.* 35 710–735. 10.1177/0890207020962330

[B41] Harari M. B. Manapragada A. Viswesvaran C. (2017). Who thinks they’re a big fish in a small pond and why does it matter? A meta-analysis of perceived overqualification. *J. Vocat. Behav.* 102 28–47. 10.1016/j.jvb.2017.06.002

[B42] Hayes A. F. (2017). *Introduction to mediation, moderation, and conditional process analysis: A regression-based approach.* New York, NY: Guilford Press.

[B43] Hobfoll S. E. (1998). *Stress, culture, and community: The psychology and philosophy of stress.* New York, NY: Plenum Press. 10.1007/978-1-4899-0115-6

[B44] Hobfoll S. E. Halbesleben J. Neveu J. P. Westman M. (2018). Conservation of resources in the organizational context: The reality of resources and their consequences. *Annu. Rev. Organ. Psychol. Organ. Behav.* 5 103–128. 10.1038/s41598-020-71501-0 32943683 PMC7498602

[B45] Höfling V. Moosbrugger H. Schermelleh-Engel K. Heidenreich T. (2011). Mindfulness or mindlessness?: A modified version of the Mindful Attention and Awareness Scale (MAAS). *Eur. J. Psychol. Assess.* 27 59–64. 10.1027/1015-5759/a000045

[B46] Hoxha S. Çetin F. (2020). The effect of psychological needs on task performance with the mediating role of work engagement: A sample from a public organization in Kosovo. *İş ve Ýnsan Dergisi* 7 1–11. 10.18394/iid.686542

[B47] Hu L.-T. Bentler P. M. (1995). “Evaluating model fit,” in *Structural equation modeling: Concepts, issues, and applications*, ed. Hoyle R. H. (Thousand Oaks, CA: Sage Publications, Inc), 76–99.

[B48] Hu L.-T. Bentler P. M. (1999). Cutoff criteria for fit indexes in covariance structure analysis: Conventional criteria versus new alternatives. *Struct. Equ. Model.* 6 1–55. 10.1080/10705519909540118

[B49] Hülsheger U. R. Walkowiak A. Thommes M. S. (2018). How can mindfulness be promoted? Workload and recovery experiences as antecedents of daily fluctuations in mindfulness. *J. Occup. Organ. Psychol.* 91 261–284. 10.1111/joop.12206 29861554 PMC5969091

[B50] Iasiello M. van Agteren J. (2020). Mental health and/or mental illness: A scoping review of the evidence and implications of the dual-continua model of mental health. *Evidence-Base J. Evid. Rev. Key Policy Areas* 1, 1–45. 10.21307/eb-2020-001

[B51] Ipsen C. Karanika-Murray M. Nardelli G. (2020). Addressing mental health and organisational performance in tandem: A challenge and an opportunity for bringing together what belongs together. *Work Stress* 34 1–4. 10.1080/02678373.2020.1719555

[B52] James L. R. Mulaik S. A. Brett J. M. (2006). A tale of two methods. *Organ. Res. Methods* 9 233–244. 10.1177/1094428105285144

[B53] Johns G. (2006). The essential impact of context on organizational behavior. *Acad. Manag. Rev.* 31 386–408. 10.2307/20159208

[B54] Johns G. (2017). Reflections on the 2016 decade award: Incorporating context in organizational research. *Acad. Manag. Rev.* 42 577–595. 10.5465/amr.2017.0044

[B55] Joo B.-K. Lee I. (2017). Workplace happiness: Work engagement, career satisfaction, and subjective well-being. *Evid. Based HRM* 5 206–221. 10.1108/EBHRM-04-2015-0011

[B56] Judge T. A. Weiss H. M. Kammeyer-Mueller J. D. Hulin C. L. (2017). Job attitudes, job satisfaction, and job affect: A century of continuity and of change. *J. Appl. Psychol.* 102 356–374. 10.1037/apl0000181 28125260

[B57] Kadir B. A. Broberg O. (2021). Human-centered design of work systems in the transition to industry 4.0. *Appl. Ergon.* 92:103334. 10.1016/j.apergo.2020.103334 33264676

[B58] Keyes C. L. M. Shmotkin D. Ryff C. D. (2002). Optimizing well-being: The empirical encounter of two traditions. *J. Pers. Soc. Psychol.* 82 1007–1022. 10.1037/0022-3514.82.6.1007 12051575

[B59] Kiken L. G. Garland E. L. Bluth K. Palsson O. S. Gaylord S. A. (2015). From a state to a trait: Trajectories of state mindfulness in meditation during intervention predict changes in trait mindfulness. *Pers. Individ. Differ.* 81 41–46. 10.1016/j.paid.2014.12.044 25914434 PMC4404745

[B60] Kline R. B. (2011). *Principles and practice of structural equation modeling*, 3rd Edn. New York, NY: Guilford Press.

[B61] Kline R. B. (2016). *Principles and practice of structural equation modeling*, 4rd Edn. New York, NY: The Guilford Press.

[B62] Lindberg C. Baranski E. Gilligan B. Fisher J. Canada K. Heerwagen J. (2021). Personality and workstation type predict task focus and happiness in the workplace. *PsyArXiv[Preprint]*. 10.31234/osf.io/nqbh9

[B63] Little T. D. Cunningham W. A. Shahar G. Widaman K. F. (2002). To parcel or not to parcel: Exploring the question, weighing the merits. *Struct. Equ. Model.* 9 151–173. 10.1207/S15328007SEM0902_1

[B64] MacKinnon D. P. Lockwood C. M. Williams J. (2004). Confidence limits for the indirect effect: Distribution of the product and resampling methods. *Multivariate Behav. Res.* 39 99–128. 10.1207/s15327906mbr3901_4 20157642 PMC2821115

[B65] Marsh H. W. Hocevar D. (1985). Application of confirmatory factor analysis to the study of self-concept: First-and higher order factor models and their invariance across groups. *Psychol. Bull.* 97:562. 10.1037/0033-2909.97.3.562

[B66] Maslach C. Leiter M. P. (2016). Understanding the burnout experience: Recent research and its implications for psychiatry. *World Psychiatry* 15 103–111. 10.1002/wps.20311 27265691 PMC4911781

[B67] Mesmer-Magnus J. Manapragada A. Viswesvaran C. Allen J. W. (2017). Trait mindfulness at work: A meta-analysis of the personal and professional correlates of trait mindfulness. *Hum. Perform.* 30 79–98. 10.1080/08959285.2017.1307842

[B68] Mohamed Osama O. Gallagher J. E. (2018). Role models and professional development in dentistry: An important resource: The views of early career stage dentists at one academic health science centre in England. *Eur. J. Dent. Educ.* 22 e81–e87. 10.1111/eje.12261 28176433

[B69] Muthén L. K. Muthén B. O. (2017). *Unusual TLI values.* Available online at: http://www.statmodel.com/download/TLI.pdf (accessed August 16, 2023).

[B70] Niinihuhta M. Häggman-Laitila A. (2022). A systematic review of the relationships between nurse leaders’ leadership styles and nurses’ work-related well-being. *Int. J. Nurs. Pract.* 28:e13040. 10.1111/ijn.13040 35102648 PMC9788052

[B71] Oplatka I. (2017). Principal workload: Components, determinants and coping strategies in an era of standardization and accountability. *J. Educ. Administr.* 55 552–568. 10.1108/JEA-06-2016-0071

[B72] Paiement A. M. Malo M. Gilbert M.-H. (2018). “La performance en emploi : Validation francophone de l’échelle de Williams et Anderson [Communication],” in *Congrès de l’Association internationale de psychologie du travail et de langue française*, France.

[B73] Park C. L. Kubzansky L. D. Chafouleas S. M. Davidson R. J. Keltner D. Parsafar P. (2023). Emotional well-being: What it is and why it matters. *Affect. Sci.* 4 10–20. 10.1007/s42761-022-00163-0 37070009 PMC10104995

[B74] Peiró J. M. Montesa D. Soriano A. Kozusznik M. W. Villajos E. Magdaleno J. (2021). Revisiting the happy-productive worker thesis from a eudaimonic perspective: A systematic review. *Sustainability* 13:3174. 10.3390/su13063174

[B75] Peiró J. Kozusznik M. Rodríguez-Molina I. Tordera N. (2019). The happy-productive worker model and beyond: Patterns of wellbeing and performance at work. *Int. J. Environ. Res. Public Health* 16:479. 10.3390/ijerph16030479 30736334 PMC6388150

[B76] Petö R. Reizer B. (2021). Gender differences in the skill content of jobs. *J. Popul. Econ.* 34 825–864. 10.1007/s00148-021-00825-6

[B77] Previtali F. Spedale S. (2021). Doing age in the workplace: Exploring age categorisation in performance appraisal. *J. Aging Stud.* 59:100981. 10.1016/j.jaging.2021.100981 34794725

[B78] Ryan R. M. Deci E. L. (2017). *Self-Determination Theory: Basic psychological needs in motivation, development, and wellness.* New York, NY: Guilford Press.

[B79] Ryff C. D. (2013). Psychological well-being revisited: Advances in the science and practice of eudaimonia. *Psychother. Psychosom.* 83 10–28. 10.1159/000353263 24281296 PMC4241300

[B80] Ryff C. D. Keyes C. L. M. (1995). The structure of psychological well-being revisited. *J. Pers. and Soc. Psychol.* 69 719–727. 10.1037/0022-3514.69.4.719 7473027

[B81] Sala M. Rochefort C. Lui P. P. Baldwin A. S. (2020). Trait mindfulness and health behaviors: A meta-analysis. *Health Psychol. Rev.* 14 345–393. 10.1080/17437199.2019.1650290 31362588

[B82] Satorra A. Bentler P. M. (2001). A sclaed difference chi-square test statistic for moment structure analysis. *Psychometrika* 66 507–514. 10.1007/BF02296192 PMC2905175 20640194

[B83] Schaufeli W. B. Bakker A. (2004). Job demands, job resources, and their relationship with burnout and engagement: A multi-sample study. *J. Organ. Behav.* 25 293–315. 10.1002/job.248

[B84] Schaufeli W. B. Salanova M. González-Romá V. Bakker A. B. (2002). The measurement of engagement and burnout: A two sample confirmatory factor analytic approach. *J. Happ. Stud.* 3 71–92. 10.1023/A:1015630930326

[B85] Seligman M. E. P. (2011). *Flourish: A visionary new understanding of happiness and well-being.* New York, NY: Free Press.

[B86] Seligman M. E. P. (2019). Positive psychology: A personal history. *Annu. Rev. Clin. Psychol.* 15 1–23. 10.1146/annurev-clinpsy-050718-095653 30525996

[B87] Sender G. Nobre G. C. Armagan S. Fleck D. (2021). In search of the Holy Grail: A 20-year systematic review of the happy-productive worker thesis. *Int. J. Organ. Anal.* 29 1199–1224. 10.1108/IJOA-09-2020-2401

[B88] Shrout P. E. Bolger N. (2002). Mediation in experimental and nonexperimental studies: New procedures and recommendations. *Psychol. Methods* 7 422–445. 10.1037/1082-989X.7.4.422 12530702

[B89] Slemp G. R. Kern M. L. Patrick K. J. Ryan R. M. (2018). Leader autonomy support in the workplace: A meta-analytic review. *Motiv. Emot.* 42 706–724. 10.1007/s11031-018-9698-y 30237648 PMC6133074

[B90] Syvertsen T. (2020). *Digital detox: The politics of disconnecting.* Boston, MA: Emerald Publishing Limited.

[B91] Syvertsen T. Enli G. (2020). Digital detox: Media resistance and the promise to authenticity. *Convergence* 26 1269–1283. 10.1177/1354856519847325

[B92] Tabachnick B. G. Fidell L. S. (2019). *Using multivariate statistics*, 7rd Edn. London: Pearson.

[B93] Toniolo-Barrios M. Pitt L. (2021). Mindfulness and the challenges of working from home in times of crisis. *Bus. Horizons* 64 189–197. 10.1016/j.bushor.2020.09.004 33041346 PMC7535863

[B94] Van den Broeck A. Ferris D. L. Chang C. H. Rosen C. C. (2016). A review of self-determination theory’s basic psychological needs at work. *J. Manag.* 42 1195–1229. 10.1177/0149206316632058

[B95] van Wingerden J. Derks D. Bakker A. B. (2018). Facilitating interns’ performance: The role of job resources, basic need satisfaction and work engagement. *Career Dev. Int.* 23 382-396. 10.1108/CDI-12-2017-0237

[B96] Williams L. L. Anderson S. E. (1991). Job satisfaction and organizational commitment as predictors of organizational citizenship and in-role behaviors. *J. Manag.* 17 601–627. 10.1177/014920639101700305

[B97] Wright T. A. Cropanzano R. (2007). The happy/productive worker thesis revised. *Res. Pers. Hum. Resour. Manag.* 26 269–307. 10.1016/S0742-7301200726

[B98] Zhao M. Y. Tay L. (2023). From ill-being to well-being: Bipolar or bivariate? *J. Posit. Psychol.* 18 649–659. 10.1080/17439760.2022.2109204

